# Potential cannabidiol (CBD) repurposing as antibacterial and promising therapy of CBD plus polymyxin B (PB) against PB-resistant gram-negative bacilli

**DOI:** 10.1038/s41598-022-10393-8

**Published:** 2022-04-19

**Authors:** Nathália Abichabki, Luísa V. Zacharias, Natália C. Moreira, Fernando Bellissimo-Rodrigues, Fernanda L. Moreira, Jhohann R. L. Benzi, Tânia M. C. Ogasawara, Joseane C. Ferreira, Camila M. Ribeiro, Fernando R. Pavan, Leonardo R. L. Pereira, Guilherme T. P. Brancini, Gilberto Ú. L. Braga, Antonio W. Zuardi, Jaime E. C. Hallak, José A. S. Crippa, Vera L. Lanchote, Rafael Cantón, Ana Lúcia C. Darini, Leonardo N. Andrade

**Affiliations:** 1 Department of Clinical Analyses, Toxicology and Food Science (DACTB), School of Pharmaceutical Sciences of Ribeirao Preto (FCFRP), University of São Paulo (USP), Av. do Café, s/nº, Campus Universitário, Ribeirão Preto, SP 14040-903 Brazil; 2 Department of Social Medicine, Ribeirão Preto Medical School (FMRP), University of São Paulo (USP), Ribeirão Preto, SP Brazil; 3 Department of Biological Sciences, School of Pharmaceutical Sciences (FCF), São Paulo State University (UNESP), Araraquara, SP Brazil; 4 Department of Pharmaceutical Sciences (DCF), School of Pharmaceutical Sciences of Ribeirão Preto (FCFRP), University of São Paulo (USP), Ribeirão Preto, SP Brazil; 5 Department of Neurosciences and Behavioral Sciences, Ribeirão Preto Medical School (FMRP), University of São Paulo (USP), Ribeirão Preto, SP Brazil; 6 National Institute of Science and Technology for Translational Medicine (INCT-TM), Conselho Nacional de Desenvolvimento Científico e Tecnológico (CNPq), Brasília, DF Brazil; 7 Hospital Universitario Ramón y Cajal and Instituto Ramón y Cajal de Investigación Sanitaria (IRYCIS), Madrid, Spain

**Keywords:** Antibiotics, Antimicrobial resistance

## Abstract

This study aimed to assess the ultrapure cannabidiol (CBD) antibacterial activity and to investigate the antibacterial activity of the combination CBD + polymyxin B (PB) against Gram-negative (GN) bacteria, including PB-resistant Gram-negative bacilli (GNB). We used the standard broth microdilution method, checkerboard assay, and time-kill assay. CBD exhibited antibacterial activity against Gram-positive bacteria, lipooligosaccharide (LOS)-expressing GN diplococcus (GND) (*Neisseria gonorrhoeae*, *Neisseria meningitidis*, *Moraxella catarrhalis*), and *Mycobacterium tuberculosis*, but not against GNB. For most of the GNB studied, our results showed that low concentrations of PB (≤ 2 µg/mL) allow CBD (≤ 4 µg/mL) to exert antibacterial activity against GNB (e.g., *Klebsiella pneumoniae, Escherichia coli, Acinetobacter baumannii*), including PB-resistant GNB. CBD + PB also showed additive and/or synergistic effect against LOS-expressing GND. Time-kill assays results showed that the combination CBD + PB leads to a greater reduction in the number of colony forming units per milliliter compared to CBD and PB alone, at the same concentration used in combination, and the combination CBD + PB was synergistic for all four PB-resistant *K. pneumoniae* isolates evaluated. Our results show that CBD has translational potential and should be further explored as a repurposed antibacterial agent in clinical trials. The antibacterial efficacy of the combination CBD + PB against multidrug-resistant and extensively drug-resistant GNB, especially PB-resistant *K. pneumoniae*, is particularly promising.

## Introduction

Antimicrobial resistance is a worldwide public health problem arising from increased incidence of bacterial health care-associated infections (HAI) caused by multidrug-resistant (MDR) and extensively drug-resistant (XDR) ESKAPE (*Enterococcus faecium*, *Staphylococcus aureus*; *Klebsiella pneumoniae*, *Acinetobacter baumannii*, *Pseudomonas aeruginosa*, and *Enterobacter* spp.) pathogens^1,2^.

Gram-negative bacilli (GNB) resistant to third- and fourth-generation cephalosporins and to carbapenem have been of particular concern, especially carbapenem-resistant *Enterobacteriaceae* (CRE; e.g., *Klebsiella pneumoniae* carbapenemase [KPC] producers), ceftazidime-avibactam-resistant (CAZ-AVI) and ceftalozane-tazobactam-resistant strains, carbapenem-resistant *A. baumannii* (CRAB), and carbapenem-resistant *P. aeruginosa* (CRPA)^3,4^.

In this context, the last-resort antibiotic polymyxin B (PB) has been used in clinical practice to treat serious infections caused by MDR/XDR GNB^5^. The antibacterial activity of polymyxins is due to an electrostatic interaction between the positively charged polymyxin and the phosphate groups of the negatively charged lipid A, on lipopolysaccharide (LPS) or lipooligosaccharide (LOS), in the outer membrane of GNB. Interaction with PB destabilizes LPS or LOS, leading to disruption of the bacterial cell envelop^3^.

However, acquired resistance to polymyxins (including chromosomal and plasmid-mediated resistance) has been increasingly detected in several GNB, such as *Enterobacterales* species (e.g., *K. pneumoniae*) and nonfermenting GNB (e.g., *A. baumannii* and *P. aeruginosa*). Polymyxin-resistant Gram-negative (GN) bacteria usually have the addition of phosphoethanolamine and/or 4-amino-4-deoxy-l-arabinose cationic groups on their lipid A molecule, giving them positive charges and resulting in electrostatic repulsion (instead of interaction) of the polymyxin molecule^3^.

Considering health, social and economic implications of growing antimicrobial resistance, the World Health Organization (WHO) calls attention to research, discovery, and development of new antibiotics against MDR/XDR ESKAPE pathogens^6^.

In this scenario, many substances have been investigated regarding their antimicrobial activity, including natural products. Cannabidiol (CBD) is the major non-psychoactive component isolated from *Cannabis sativa* and has been associated with multiple and potential biological activities, especially anxiolytic, antipsychotic, anti-inflammatory, analgesic, antioxidant and neuroprotective properties^7–9^. Regarding its biological activity in bacteria, CBD was described as inhibitor of membrane vesicles released from GNB and as inhibitor of biofilm formation as well as being capable of eradicating preformed biofilms^10^. Since the 1950s, *C. sativa*-based preparations have also been investigated for their antibacterial activity^11–13^. Nonetheless, few studies described the antibacterial activity of ultrapure CBD against Gram-positive (GP) bacteria; although, CBD is not antibacterial against GNB^9,11–17^.

Another promising strategy to tackle antibacterial resistance is combination therapy, specifically combinations that allow the use of a lower concentration of each substance in the combination or that allow the antibacterial activity of substances that would otherwise not reach the bacterial target (e.g., due to impermeability)^18,19^. Besides their inhibitory activity, antibiotics can exert other effects on bacterial cells, even at sublethal concentrations^20^. In this context, the minimal effective antibiotic concentration (MEAC), defined as the minimal sublethal concentration that produces an effect on bacterial cells (e.g., outer membrane destabilization), might allow or improve the action of another antibacterial substance, thus making the combination antibacterial^21,22^.

The present study aimed to evaluate the in vitro antibacterial activity of ultrapure CBD against a wide diversity of bacteria, including MDR/XDR ESKAPE pathogens (44 different species, 96 strains), and the in vitro antibacterial activity of the combination CBD + PB against GN bacteria, including PB-susceptible and PB-resistant GNB (chromosomal-acquired and plasmid-mediated colistin-resistant) and intrinsically-resistant GN bacteria (16 species, 56 strains), comprising both standard strains and clinical isolates.

## Results

### CBD antibacterial activity against GP and GN bacteria, and *Mycobacterium tuberculosis*

CBD showed different levels of antibacterial activity (minimal inhibitory concentration [MIC]) against all 13 different species of GP bacteria (21 strains) evaluated, including susceptible and MDR strains: MIC = 2 µg/mL for *E. faecium* (n = 2); MIC = 4 µg/mL for *Enterococcus* spp. (n = 4), *Staphylococcus* spp. (n = 10), *Micrococcus luteus* (n = 1), and *Rhodococcus equi* (n = 1); MIC = 32 µg/mL for *Streptococcus pyogenes* (n = 1) and *Streptococcus pneumoni*ae (n = 1); and MIC = 64 µg/mL *Streptococcus agalactiae* (n = 1) (Supplementary Table 1).

The antibacterial activity of CBD was also observed for LOS-expressing GN diplococcus (GND) such as *Moraxella catarrhalis* ATCC 25238 (MIC = 64 µg/mL), *Neisseria meningitidis* ATCC 13077 (MIC = 128 µg/mL)*,* and *Neisseria gonorrhoeae* ATCC 19424 (MIC = 256 µg/mL). Additionally, CBD was antibacterial against *Mycobacterium tuberculosis* H37Rv (MIC = 12.5 µg/mL) and MDR *M. tuberculosis* CF86 (MIC = 25 µg/mL). We observed no difference between MIC and minimum bactericidal concentration (MBC) values among susceptible and MDR strains evaluated.

For *S. aureus* ATCC 29213, we observed a higher CBD MIC (64 µg/mL) when the assay was performed using MH-F broth (5% lysed horse blood + 0.1% β-Nicotinamide adenine dinucleotide [β-NAD] 20 mg/mL), compared to standard protocols using Cation-Adjusted Mueller Hinton II Broth (CAMHB) for *S. aureus* (CBD MIC = 4 µg/mL) (Supplementary Fig. 1)^23,24^.

CBD in concentrations up to 256 µg/mL was not antibacterial for any of the tested GNB (27 species, 70 strains) (Supplementary Table 1). We also evaluated higher concentrations of CBD for *E. coli* ATCC 25922, *K. pneumoniae* ATCC 13883, *A. baumannii* ATCC 19606, and *P. aeruginosa* ATCC 27853, but again no antibacterial activity was observed up to 8.192 µg/mL.

Additionally, we did not detect any antibacterial activity of CBD in the presence of efflux pump inhibitors (phenylalanine-arginine-β-naphthylamide [PAβN], reserpine, or curcumin) against Gram-negative ESKAPE pathogens (*K. pneumoniae* ATCC 13883*, A. baumannii* ATCC 19606*, P. aeruginosa* ATCC 27853*, E. cloacae* ATCC 13047) as well as against *E. coli* ATCC 25922 and 72H (MCR-1-producing clinical isolate) and *S. maltophilia* ATCC 13637 strains. For all strains, CBD combined to the efflux pump inhibitors evaluated was not antibacterial up to 256 µg/mL of CBD.

### Antibacterial activity of CBD in combination with PB (CBD + PB) against GN bacteria

#### Screening by broth microdilution method with fixed concentration of CDB (256 µg/mL)

We observed antibacterial activity of the combination CBD + PB against 8/13 different species (47/52 strains) of GNB, including standard strains (Table 1) and clinical isolates (Table 2).

**Table 1:** PB concentrations in the combination of CBD (256 µg/mL) + PB minimal effective antibiotic concentration (MEAC), compared to PB, MIC against standard strains, type strains, and characterized strains.

Strain	PB (µg/mL)		Strain characteristics; references
	MEAC in the combination (CBD + PB)	MIC	
*K. pneumoniae* ATCC 13883^T^	0.125	8	Type strain
*K. pneumoniae* ATCC BAA-1705	0.25	0.5	ST 258; CRE/CPE, KPC-2, PB-susceptible
*K. pneumoniae* NCTC 13443	0.06	1	CRE/CPE, NDM, PB-susceptible
*K. pneumoniae* C9	≤ 1	64	ST 11; ESBL, CTX-M-2; PB-resistant [Δ *mgr*]; Palmeiro et al.^25^
*K. pneumoniae* D1	0.25	1	ST 11; ESBL, CTX-M-2; Palmeiro et al.^25^
*K. pneumoniae* RP 62	≤ 0.5	32	ST 11; CRE/CPE, KPC-2; Andrade et al.^26^
*K. quasipneumoniae* ATCC 700603	0.125	0.5	ESBL, SHV-18
*E. cloacae* ATCC 13047T	≤ 0.5	256	Type strain
*E. coli* ATCC 25922	0.06	0.5	Quality control strain, PB-susceptible
*E. coli* CTX-M-15	0.06	0.5	ST 131; ESBL, CTX-M-15, PB-susceptible
*E. coli* RP62T^†^	≤ 0.01	2	Transconjugant azide-resistant producing KPC, PB-susceptible; Andrade et al.^26^
*E. coli* 72H	1	2	Plasmid-mediated colistin-resistant (MCR-1); Fernandes et al.^27^
*E. coli* NCTC 13846	1	2	Plasmid-mediated colistin-resistant (MCR-1), Quality control strain
*A. baumannii* ATCC 19606^T^	0.25	1	Type strain, PB-susceptible
*A. baumannii* 136 SP	0.25	1	ST 109; CRAB, OXA-23 and OXA-143; Clímaco et al.^28^, PB-susceptible
*P. aeruginosa* ATCC 27853	1	1	Quality control strain, PB-susceptible
*P. aeruginosa* HC 103	1	2	ST 277; SPM-1, PB-susceptible; Galetti et al.^29^
*P. aeruginosa* PAO1	0.5	1	PB-susceptible
*S. maltophilia* ATCC 13673^T^	0.25	2	Type strain, PB-susceptible
*E. tarda* ATCC 15947^T^	0.25	> 8	Intrinsically PB-resistant
*S. marcescens* ATCC 13880	> 8	> 8	Intrinsically PB-resistant
*P. rettgeri* ATCC 29944	> 8	> 8	Intrinsically PB-resistant
*P. mirabilis* ATCC 29906	> 8	> 8	Intrinsically PB-resistant
*B. cepacia* ATCC 25416	> 8	> 8	Intrinsically PB-resistant
*M. morganii* ATCC 8019	> 8	> 8	Intrinsically PB-resistant

*CBD* Cannabidiol, *PB* Polymyxin B, *ATCC American Type Culture Collection*, ^T^*type strain*, *ST* sequence type, *CRE* carbapenem-resistant *Enterobacteriaceae, CPE* carbapenemase-producing *Enterobacteriaceae, KPC Klebsiella pneumoniae* carbapenemase, *NDM* New Delhi metallo-beta-lactamase (carbapenemase), *ESBL* extended spectrum beta-lactamase, *CTX-M* Active on cefotaxime, first isolated at Munich, *SHV* Sulfhydryl reagent variable, *MCR* plasmid-mediated colistin resistant, *CRAB* carbapenem resistant *Acinetobacter baumannii, OXA* Oxacilinase (OXA-23 and OXA-143 are carbapenemases), *SPM* São Paulo Metallo-beta-lactamase (carbapenemase).

**Table 2:** PB concentrations in the combination of CBD (256 µg/mL) + PB minimal effective antibiotic concentration (MEAC) compared to PB minimal inhibitory concentration (MIC) against clinical isolates.

Strain	PB (µg/mL)		Strain characteristics^a^
	MEAC in the combination (CBD + PB)	MIC	
*K. pneumoniae* L1	≤ 0.25	8	Clinical isolate; PB-resistant
*K. pneumoniae* L2	≤ 0.5	64	Clinical isolate; PB-resistant
*K. pneumoniae* L3	≤ 0.25	64	Clinical isolate; PB-resistant
*K. pneumoniae* L5	≤ 0.03	1	Clinical isolate; PB-susceptible
*K. pneumoniae* L8	≤ 0.25	128	Clinical isolate; PB-resistant
*K. pneumoniae* L9	≤ 0.25	32	Clinical isolate; PB-resistant
*K. pneumoniae* L12	0.06	2	Clinical isolate; PB-susceptible; CAZ-AVI-resistant
*K. pneumoniae* L13	≤ 0.25	16	Clinical isolate; PB-resistant
*K. pneumoniae* L14	≤ 0.25	32	Clinical isolate; PB-resistant
*K. pneumoniae* L15	0.5	4	Clinical isolate; PB-resistant
*K. pneumoniae* L16	0.5	4	Clinical isolate; PB-resistant
*K. pneumoniae* L17	≤ 0.5	32	Clinical isolate; PB-resistant
*K. pneumoniae* L18	0.5	4	Clinical isolate; PB-resistant
*K. pneumoniae* L19	1	16	Clinical isolate; PB-resistant
*K. pneumoniae* L22	≤ 0.5	256	Clinical isolate; PB-resistant
*K. pneumoniae* L26	≤ 0.5	64	Clinical isolate; PB-resistant
*K. pneumoniae* L27	0.06	2	Clinical isolate; PB-susceptible
*K. pneumoniae* L28	≤ 0.5	32	Clinical isolate; PB-resistant
*K. pneumoniae* L29	≤ 0.25	16	Clinical isolate; PB-resistant
*K. pneumoniae* L30	0.25	1	Clinical isolate; PB-resistant
*K. pneumoniae* L31	0.06	8	Clinical isolate; PB-resistant
*K. pneumoniae* L33	0.06	16	Clinical isolate; PB-resistant
*K. pneumoniae* L34	≤ 0.125	32	Clinical isolate; PB-resistant
*A. baumannii* L7	≤ 0.03	2	Clinical isolate; PB-susceptible
*A. baumannii* L21	0.125	1	Clinical isolate; PB-susceptible
*A. baumannii* L25	0.125	1	Clinical isolate; PB-susceptible
*A. baumannii* L35	0.125	0.5	Clinical isolate; PB-susceptible
*P. aeruginosa* L36	1	2	Clinical isolate; PB-susceptible

*CBD* Cannabidiol, *PB* Polymyxin B, *CAZ-AVI* Ceftazidime-avibactam.

^a^PB-resistant clinical isolates are not plasmid-mediated colistin-resistant (MCR-1).

For the combination CBD (256 µg/mL) + PB (0.01—512 µg/mL), compared to PB alone, we observed a minimal threefold reduction in the PB concentration required for CBD antibacterial activity against PB-susceptible GNB (non-fermenting GNB and *Enterobacterales*). Also, the combination of CBD + PB against *K. pneumoniae* led to a twofold reduction in PB concentration compared to PB MIC, while only a onefold reduction was observed for *P. aeruginosa* (Tables 1 and 2, and Supplementary Fig. 2A).

Regarding PB-resistant GNB (*Enterobacterales*), the combination of CBD plus low concentrations of PB (≤ 2 µg/mL) showed antibacterial activity against chromosomal PB-resistant GNB, including PB-resistant *K. pneumoniae* (Table 2). However, for plasmid-mediated colistin-resistant (MCR-1) *E. coli* strains, we only observed a onefold reduction in PB concentration compared to PB MIC (Tables 1 and 2, and Supplementary Fig. 2B–F).

The combination CBD + PB + PAβN showed increased antibacterial activity against GNB so that lower concentrations of PB were required when compared to the combination CBD + PB (Table 3). Interestingly, this combination (CBD + PB + PAβN) also showed antibacterial activity against *P. aeruginosa* and plasmid-mediated colistin-resistant (MCR-1) *E. coli* 72H strains (Table 3).

**Table 3:** PB concentrations in the combination of CBD (256 µg/mL) + PB + PAβN (50 µg/mL) compared to PB concentrations in the combination of CBD (256 µg/mL) + PB.

Strain	PB MIC (µg/mL)	CBD 256 µg/mL	CBD 256 µg/mL plus PAβN 50 µg/mL	PAβN 50 µg/mL
		+ PB MEAC (µg/mL)	+ PB MEAC (µg/mL)	+ PB MEAC (µg/mL)
*K. pneumoniae* ATCC 13883^a^	8	0.125	≤ 0.01	8
*K. pneumoniae* ATCC BAA-1705	0.5	0.25	0.01	0.25
*K. quasipneumoniae* ATCC 700603	0.5	0.125	0.03	0.25
*E. coli* ATCC 25922	0.5	0.06	≤ 0.002	0.03
*E. coli* 72H^b^	2	1	≤ 0.01	1
*E. coli* NCTC 13846	2	1	≤ 0.03	1
*A. baumannii* ATCC 19606^a^	1	0.25	≤ 0.005	1
*A. baumannii* 136 SP^c^	1	0.25	0.06	1
*P. aeruginosa* ATCC 27853	1	1	≤ 0.005	0.06
*P. aeruginosa* HC 103^j^	2	1	≤ 0.005	0.06
*P. aeruginosa* PAO1	1	0.5	≤ 0.005	0.06
*S. maltophilia* 13673^a^	2	0.25	≤ 0.01	0.03
*E. tarda* ATCC 15947^a^	> 8	0.25	≤ 0.01	> 8

*CBD* Cannabidiol, *PB* Polymyxin B, *ATCC* American Type Culture Collection.

^a^Type strain.

^b^Fernandes et al.^27^.

^c^Clímaco et al.^30^.

^d^Galetti et al.^29^.

CBD + PB also presented antibacterial activity against intrinsically PB-resistant *E. tarda* ATCC 15947 (Table 1). Nevertheless, for other intrinsically PB-resistant strains (*B. cepacia* ATCC 25416, *M. morganii* ATCC 8019, *P. rettgeri* ATCC 29944, *P. mirabilis* ATCC 29906, and *S. marcescens* subsp. *marcescens* ATCC 13880), no antibacterial activity of CBD + PB was observed (Table 1).

Regarding intrinsically PB-resistant GNB, the combination of CBD + PB was not antibacterial even in the presence of PAβN. The exception was *E. tarda* ATCC 15947, for which the combination CBD + PB + PAβN was also antibacterial, again showing the antibacterial activity of CBD with lower PB concentrations (Table 3).

#### Confirmation by checkerboard assay

For *K. pneumoniae* (n = 12), *E. coli* (n = 4), *A. baumannii* (n = 2), and *P. aeruginosa* (n = 2), checkerboard assay was performed to confirm the in vitro antibacterial activity and to assess the different proportions of each substance in the combination CBD + PB (Table 4).

**Table 4:** Minimal concentrations of CBD and PB (PB minimal effective antibiotic concentration [MEAC]) required to the antibacterial activity of the combination CBD + PB, according to checkerboard assay results. Plasmid-mediated colistin-resistant (MCR-1) *E. coli* 72H and *P. aeruginosa* strains (ATCC 27853 and HC 103) were assessed in the presence of 50 µg/mL of PAβN.

Strain	Antibacterial activity of the combination CBD + PB (µg/mL)		PB MIC (µg/mL)	PB MIC versus PB MEAC (two-fold differences)
	CBD	PB MEAC		
*K. pneumoniae* ATCC 13883^a^	4	0.03	8	8
*K. pneumoniae* ATCC BAA-1705	4	0.25	0.5	1
*K. pneumoniae* C9^b^	4	2	64	5
*K. pneumoniae* D1^b^	4	0.25	1	2
*K. pneumoniae* RP 62^c^	2	1	32	6
*K. pneumoniae* L8	2	≤ 1	128	8
*K. pneumoniae* L13	2	≤ 0.25	32	8
*K. pneumoniae* L17	4	2	32	5
*K. pneumoniae* L22	2	≤ 0.5	256	10
*K. pneumoniae* L28	2	≤ 0.5	32	7
*K. pneumoniae* L29	2	≤ 0.5	32	7
*K. pneumoniae* L34	2	0.5	32	7
*E. coli* ATCC 25922	4	0.06	0.5	3
*E. coli* CTX-M-15	8	0.03	0.5	4
*E. coli* 72H^d^ (MCR-1)	4 + **PAβN**	1	2	1
*E. coli* NCTC 13846 (MCR-1)	4 + **PAβN**	1	2	1
*A. baumannii* ATCC 19606^a^	4	0.125	1	3
*A. baumannii* 136 SP^e^	1	0.25	1	2
*P. aeruginosa* ATCC 27853	4 + **PAβN**	0.03	1	5
*P. aeruginosa* HC 103^f^	4 + **PAβN**	0.03	2	6

*ATCC* American Type Culture Collection.

^a^Type strain.

^b^Palmeiro et al.^25^.

^c^Andrade et al.^26^.

^d^Fernandes et al.^27^.

^e^Clímaco et al.^30^.

^f^Galetti et al.^29^.

For most of the GNB (including PB-resistant *K. pneumoniae),* 2–4 µg/mL of CBD were enough to inhibit bacterial growth when combined with low concentrations of PB (≤ 2 µg/mL) (Table 4). Particularly, the combination of ≤ 2 µg/mL of CBD plus ≤ 0.5 µg/mL of PB was antibacterial to most PB-resistant clinical isolates of *K. pneumoniae* (Supplementary Fig. 3).

For PB-susceptible *P. aeruginosa* ATCC 27853 and HC103, and plasmid-mediated colistin-resistant (MCR-1) *E. coli* 72H strains, the checkerboard assay was also performed in the presence of PAβN. The results showed that the combination of CBD (4 µg/mL) + PB was antibacterial only in the presence of PAβN (Table 4).

The fractional inhibitory concentration index (FICI) of the combination of CBD + PB was not calculated due to the absence of antibacterial activity (MIC) of CBD against GNB.

For GND *M. catarrhalis* ATCC 25238, *N. meningitidis* ATCC 13077*,* and *N. gonorrhoeae* ATCC 19424, the FICI of the combination CBD + PB was calculated because both CBD and PB alone showed antibacterial activity (MIC). Thereby, CBD + PB showed additive and/or synergistic effect against these GND (Table 5, and Supplementary Fig. 4).

**Table 5:** Minimal concentrations of CBD and PB (PB minimal effective antibiotic concentration [MEAC]) required to the antibacterial activity of the combination CBD + PB. Fractional Inhibitory Combination Index (FICI) values, according to checkerboard assay results. CBD showed antibacterial activity against GND, so FICI was calculated, and the effect of the combination was characterized.

Strain	CBD MIC (µg/mL)	Antibacterial activity of the combination CBD + PB (µg/mL)		PB MIC (µg/mL)	FICI	Effect
		CBD	PB MEAC			
*M. catarrhalis* ATCC 25238	64	4	0.25	0.5	0.56	Additive
		16	0.01		0.27	Synergic
*N. meningitidis* ATCC 13077	128	32	2	64	0.28	Synergic
		16	8		0.25	
		8	16		0.31	
*N. gonorrhoeae* ATCC 19424	256	32	0.5	16	0.155	Synergic
		16	4		0.31	

### Time-kill assays

**Figure 1:**
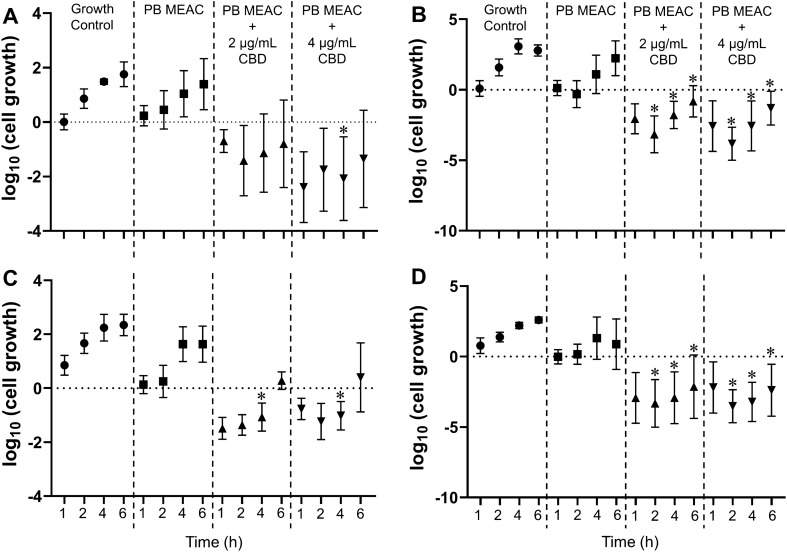
Time-kill experiments for the PB-resistant *Klebsiella pneumoniae* strains (**A**) C9, (**B**) L8, (**C**) L28, and (**D**) L29. Strains were inoculated on Mueller Hinton broth containing either PB alone or in combination with CBD (2 or 4 µg/mL). A control group was grown in the absence of any drug. At time points 0, 1, 2, 4, and 6 h, an aliquot was removed, and number of cells was determined by inoculating on solid medium and counting colony forming units. The initial time point of each treatment was used as normalization factor. Values represent mean and error bars are standard deviation of the mean from three independent experiments. Asterisks indicate that means are statistically different from the corresponding control group (*P* < 0.05). PB: polymyxin B; CBD: cannabidiol; MEAC: minimal effective antibiotic concentration.

Time-kill assays showed that the combination CBD + PB leads to a greater reduction in the number of CFU/mL compared to PB alone (at the same concentration used for the combination) for all four clinical isolates of PB-resistant *K. pneumoniae* evaluated (Fig. 1). Also, the overall reduction in CFU/mL of the combination CBD + PB relative to PB alone was above 2 log_10_ for many time points, further confirming the synergistic effect of CBD and PB (Table 6).

**Table 6:** Log_10_ (CFU/mL) difference between the combination CBD + PB and PB treatment at MEAC.

Strain	CBD = 2 µg/mL				CBD = 4 µg/mL			
	Kill_1h_	Kill_2h_	Kill_4h_	Kill_6h_	Kill_1h_	Kill_2h_	Kill_4h_	Kill_6h_
*K. pneumoniae* C9	− 0.93	− 1.87	**− 2.18**	**− 2.19**	**− 2.63**	**− 2.20**	**− 3.12**	**− 2.74**
*K. pneumoniae* L8	**− 2.18**	**− 2.85**	**− 2.88**	**− 3.05**	**− 2.70**	**− 3.52**	**− 3.66**	**− 3.54**
*K. pneumoniae* L28	− 1.62	− 1.61	**− 2.70**	− 1.35	− 0.90	− 1.48	**− 2.65**	− 1.23
*K. pneumoniae* L29	**− 2.92**	**− 3.49**	**− 4.22**	**− 3.01**	**− 2.17**	**− 3.69**	**− 4.52**	**− 3.27**

Combinations were considered synergistic (indicated by bold) when the difference was greater (more negative) than 2 log_10_ compared to the most active component of the combination (i.e., PB).

## Discussion

### CBD antibacterial activity against GP and GN bacteria, and *Mycobacterium tuberculosis*

We observed antibacterial activity of ultrapure CBD against GP bacteria, *M. tuberculosis*, and LOS-expressing GND; but not against GNB as recently reported^14–16^. Preliminary results regarding ultrapure CBD activity in GP bacteria and its absence in GN bacteria (including MDR and XDR strains) were partially presented by us at ASM Microbe 2018 and published in the abstract book^13^.

Furthermore, our results revealed antibacterial activity of CBD against other species, namely *Enterococcus casseliflavus, Staphylococcus lugdunensis, Micrococcus luteus,* and *Rhodococcus equi*, and also against different phenotype/genotype of GP bacteria (Supplementary Table 1). In general, our data and previous reports showed CBD MICs ranging from 2 to 4 µg/mL against GP bacteria, including vancomycin-resistant *E. faecium* (VRE), and methicillin-resistant (MRSA) and vancomycin-intermediate resistant (VISA) *S. aureus*, all of them listed in the WHO priority pathogens list for R&D of new antibiotics^16^.

Our data differ from the data by Blaskovich et al. on CBD MICs against *S. pneumoniae, S. pyogenes, N. meningitidis, N. gonorrhoeae, M. catarrhalis,* and *M. tuberculosis*^16^. Differences in observed MIC values may be related to the different methodology in each of the studies. For fastidious bacteria, such as *S. pneumoniae, S. pyogenes, M. catarrhalis,* and *N. meningitidis*, we used MH-F broth (CAMHB supplemented with 5% lysed horse blood + 0.1% β-NAD 20 mg/mL), according to a standard protocol by the European Committee on Antimicrobial Susceptibility Testing (EUCAST)^31^. For *N. gonorrhoeae*, we employed the broth microdilution method instead of agar dilution. Blaskovich et al*.* used a broth culture medium composed of a lower content of lysed horse blood (3%) for *S. pneumoniae* and *S. pyogenes*, and a modified broth according to standards from the American Type Culture Collection (ATCC) for *Neisseria* spp., which does not contain blood^16^. The presence of blood in culture media (e.g., MH-F broth) increases CBD MIC, as observed in our study for *S. aureus* (fourfold dilution increased CBD MIC) when compared to only CAMHB, a result similar to previous reports^12^. These differences may have contributed to our higher CBD MIC values against fastidious bacteria compared to those of Blaskovich et al.^16^.

For *M. tuberculosis*, we observed lower CBD MIC values than those of Blaskovich et al. They used a 5-day incubation period , the addition of 12.5uL of 20% Tween 80 into resazurin, and a culture medium supplemented with ADC (albumin, dextrose, catalase) (Difco Laboratories), 0.5% glycerol, and 0.02% tyloxapol^16^. We used a period of incubation of 7 days, no Tween 80 into resazurin, a culture medium supplemented with OADC (oleic acid, albumin, dextrose, catalase), and glycerol 0,4%. The difference between OADC and ADC is the presence of oleic acid at 0.5 g/L.

Our results reinforce that CBD alone is not antibacterial against GNB (MDR/XDR or susceptible to antibiotics), because we evaluated 27 species (70 strains) of GNB species most commonly involved in HAI as well as in community infections, expanding the panel of GNB and human pathogens investigated (Supplementary Table 1).

In an attempt to understand this lack of effect, we used various efflux pump inhibitors to potentially improve CBD activity. However, our data also showed no role of efflux pumps that are commonly involved in antibiotic extrusion from GN cell. Therefore, we hypothesized that CBD was inactive due to low permeability through the cell envelope (outer membrane) of GNB.

The absence of antibacterial activity of CBD against GNB may be related to LPS molecules and outer membrane proteins, from the outer membrane, which would lead to the impermeability of macromolecules and limited diffusion of hydrophobic molecules, such as CBD^14–16^. Our results of in vitro CBD antibacterial activity against GP bacteria and *M. tuberculosis*, and the absence of CBD antibacterial activity against GNB, support a role for LPS in hindering CBD activity.

Furthermore, we also assessed LOS-expressing bacteria such as *N. meningitidis*, *N. gonorrhoeae,* and *M. catarrhalis*. The LOS molecule lacks the O-antigen of LPS^32,33^, thereby allowing us to evaluate a potential role of O-antigen in preventing CBD antibacterial activity (possibly due to the steric effect, hindering CBD from reaching its molecular target).

Even considering that *A. baumannii* and *H. influenzae* also have LOS molecules on their external membrane, but the core polysaccharide for these bacteria presents a different sugar composition^32,34^. This fact could explain the absence of CBD antibacterial activity against these GN bacteria. CBD and cannabigerol (CBG, another cannabinoid) have antibacterial activity against *A. baumannii* only in the absence of the complete LOS molecule, according to previous studies^14,16^.

The hydrophobic chemical structure of CBD points towards an interaction with lipid in membranes as described by Guard et al. for eukaryotic cells. This interaction alters the biophysical properties of the membrane and affects lipid and cholesterol metabolism^35,36^. Indeed, the bacterial membrane was also suggested as a possible bacterial target for cannabinoids (CBG and CBD)^11,14,16^. Furthermore, Blaskovich et al. also showed that bactericidal concentrations of CBD against *S. aureus* inhibits the synthesis of proteins, DNA, RNA, and peptidoglycan^16^. Nevertheless, the specific mechanism(s) for the antibacterial activity of CBD has not yet been fully elucidated. Therefore, our results contribute to a better understanding of CBD antibacterial mechanism(s) of action, which could guide future studies.

### Antibacterial activity of the combination CBD + PB

Previous studies reported the antibacterial activity of CBD in combination with PB, although only few bacterial species and strains were evaluated (*E. coli*, *P. aeruginosa*, *K. pneumoniae,* and *A. baumannii*)^14,16,17,37^*.*

Here we assessed the combination CBD + PB against several GNB (8 species, 48 strains), focusing on pathogens from the WHO list for the R&D of new antibiotics, including MDR and XDR international high-risk clones (e.g., KPC-producing *K. pneumoniae* ST 258 and ST 11, CTX-M-15-producing *E. coli* ST 131, SPM-producing *P. aeruginosa* ST 277, and OXA-143-producing *A. baumannii* ST 109) (Tables 1 and 2).

For most GNB, our checkerboard results showed that CBD concentrations lower than 4 µg/mL were sufficient for antibacterial activity in the combination CBD + PB. Also, PB concentrations needed for the combination to be antibacterial were up to eight-fold lower than the MIC for PB.

Furthermore, for PB-resistant GNB (highlighting *K. pneumoniae* strains), 2–4 µg/mL of CBD were enough to lead to bacterial growth inhibition when combined with clinically optimal PB concentrations (Table 4).

According to EUCAST/BrCAST breakpoints, bacteria presenting PB MIC ≤ 2 µg/mL are categorized as susceptible, and there is a high likelihood of therapeutic success using a standard dosing regimen of PB^23,38^. On the other hand, according to CLSI breakpoints, bacteria with PB MIC ≤ 2 µg/mL are categorized as intermediate as there is no longer a CLSI ‘susceptible’ category for polymyxins. CLSI argues that polymyxins monotherapy would have limited clinical efficacy and suggests combination therapy with another antibacterial^24^. In our study, we used the EUCAST/BrCAST breakpoint (PB MIC ≤ 2 µg/mL as susceptible) for our analyses and discussion.

Additionally, time-kill results showed the killing effect of the combination of CBD + PB over time, contributing to future studies and perspectives on dose-exposure response relationships and pharmacokinetic/pharmacodynamic parameters.

Among intrinsically PB-resistant GNB, the combination of CBD + PB showed antibacterial activity only against *E. tarda*. These results may be related to the different intrinsic resistance mechanisms of these bacteria, involving different molecular pathways from two-component systems^3,39^.

For the GND *N. meningitidis*, *N. gonorrhoeae,* and *M. catarrhalis,* the calculation of FICI revealed an additive or synergistic effects for the combination CBD + PB (Table 5). Nevertheless, PB is not used for the treatment of GND-caused infections due to PB intrinsic resistance. However, the synergistic effect may suggest a new insight for this bactericidal activity of the combination CBD + PB, highlighting that PB also neutralizes the endotoxin Lipid A from LPS/LOS of GN bacteria^40^.

### Biological activity of the combination CBD + PB (+ PAβN)

CBD alone shows antibacterial activity against GP bacteria, *M. tuberculosis*, and LOS-expressing GND; however, it does not show antibacterial activity against GNB, probably due to the presence of LPS molecules and outer membrane proteins, from the outer membrane, resulting in impermeability of CBD.

PB alone shows antibacterial activity against GN bacteria and the use of this antibiotic in clinical practices depends on in vitro susceptibility breakpoints and on bacterial species identified^23,24,38^. PB promotes the destabilization of LPS or LOS, leading to the disruption of the bacterial cell envelope^3^.

Considering the antibacterial activity of the combination CBD + PB against GNB, our results also point to the existence of a CBD molecular target in GNB and indicate that its activity is dependent on bacterial outer membrane destabilization promoted by PB.

**Figure 2:**
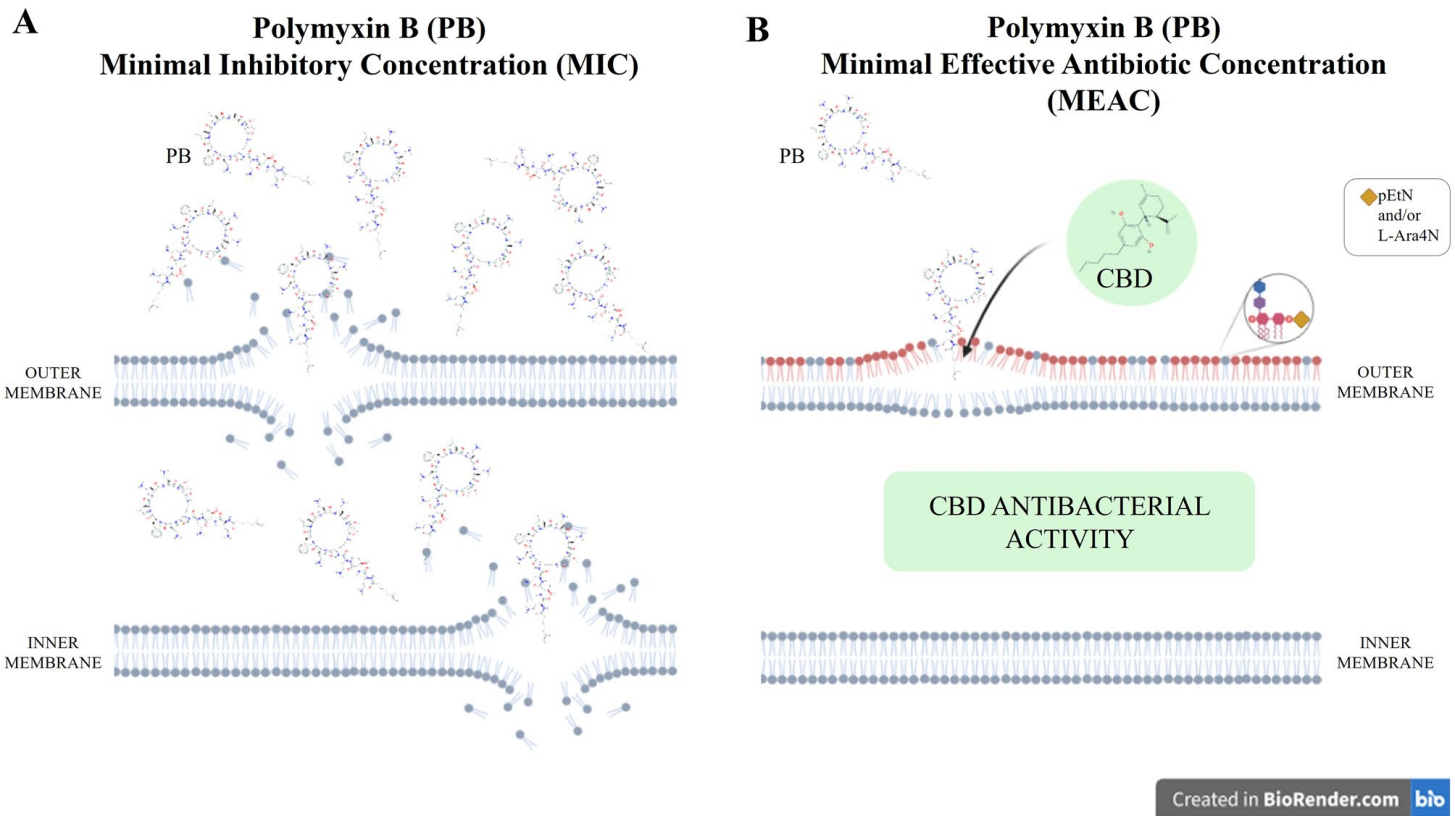
Schematic representation of PB MIC and PB MEAC. (**A**) PB MIC: Polymyxin B minimal concentration that disrupts the outer and inner membranes of GNB and leads to bacterial growth inhibition. (**B**) PB MEAC: Polymyxin B sub-inhibitory concentration that leads to minimal disrupt or destabilization of bacterial outer membrane but does not lead to bacterial growth inhibition. PB MEAC allows CBD antibacterial activity in the combination CBD + PB, even against PB-resistant strains containing phosphoethanolamine (pEtN) and/or 4-amino-4-deoxy-l-arabinose (l-Ara4N) cationic groups on their lipid A molecule.

CBD is the antibacterial agent in the combination CBD + PB, considering that the concentrations of PB used in the combination were PB MEAC, which are sublethal (subinhibitory) (Fig. 2). This biological activity is supported by time-kill results showing that PB MEAC alone has the same behavior as the growth control in the killing curve (Fig. 1).

Outstandingly, the combination CBD + PB was effective against PB-resistant *K. pneumoniae* (PB MIC ranged from 4 to 256 µg/mL), considering PB MIC > 2 µg/mL for PB-resistant strains. This fact further points towards an antibacterial activity for CBD, once PB MEAC (sublethal concentrations) were used in the combination CBD + PB.

Thereby, CBD does not restore PB susceptibility for PB-resistant GN bacteria. PB at MEAC acts exclusively as an outer membrane destabilizing agent and does not lead to bacterial cell disruption and death.

Indeed, CBD does not decrease the PB MIC against bacteria, since ‘MIC’ is the ‘minimal inhibitory concentration’ of only one antibacterial agent, so, in combination with another substance, the concept of MIC reduction is mistaken and should not be used. The lower PB concentrations of PB in the combination CBD + PB are the PB MEAC.

Furthermore, as a CBD MIC could not be determined for GNB, the antibacterial activity of the combination CBD + PB could not be categorized as synergistic or additive based on checkerboard assay due to the lack of a mathematical factor (MIC) to calculate the FICI. In addition, the attribution of the maximum concentration evaluated in the experiments as a ‘MIC’ is also a mistake.

Considering the “lack of antibacterial activity (or lack of MIC) of one substance in the drug combination”, the association of checkerboard and time-kill assays contributes to a better characterization of the combined antibacterial activity of the two substances. Checkerboard assays show how much drugs concentrations can be decreased while inhibiting bacterial growth, whereas time-kill assays show how much more effective is the combination when compared to each substance alone. Checkerboard data of combination inhibition are important; however, time-kill data are more suitable to categorize the combination effect as synergistic^41^.

Our results showed that low concentrations of PB (lower than the PB MIC against each evaluated GNB) are sufficient to cause the minimal outer membrane destabilization required to allow antibacterial activity of CBD in GNB (except *P. aeruginosa* and plasmid-mediated colistin-resistant [MCR-1] *E. coli* strains) (Table 3).

The combination CBD + PB + PAβN was effective against *P. aeruginosa* and plasmid-mediated colistin-resistant (MCR-1) *E. coli* strains, for which only CBD + PB was not active. However, these results could not be related to efflux inhibition by PAβN per se, because CBD antibacterial activity alone was not detected in the presence of PAβN. Thereby, our results suggest PAβN permeabilization of the outer membrane contributing to CBD activity, as similarly described for β-lactams in the presence of PAβN against *P. aeruginosa*, or sensitization of *P. aeruginosa* to antibiotics (e.g., vancomycin) that are typically incapable of crossing the outer membrane^42^. Indeed, the combination CBD + PB in the presence of PAβN decreases considerably the PB concentrations necessary to allow CBD activity (Table 4).

Even though PAβN is a substance commonly used for bacterial efflux pump inhibition in in vitro assays, it is not currently used as a drug in clinical practice. However, a potential use of PAβN as an antibiotic adjuvant that can reduce the effective doses of drugs that require increased outer membrane permeability has been considered^42^.

### Pharmacokinetic perspectives of cannabidiol repurposing as antibacterial

In 2018, the Food and Drug Administration (FDA, United States) approved CBD (Epidiolex^®^, Greenwich Biosciences, Inc.) for the treatment of patients with Lennox-Gastaut syndrome or Dravet syndrome^43^. Similarly, according to the European Medicines Agency, Epidyolex^®^ (GW Pharma [International] B.V.) CBD was authorized in the European Union in 2019 for the same therapeutic indication in addition to clobazam, another antiepileptic drug^44^. In 2020, the Brazilian Health Regulatory Agency (ANVISA) approved CBD (Canabidiol Prati-Donaduzzi^®^, Toledo, PR, Brazil) for the treatment of pharmacoresistant epilepsy or refractory epilepsy^45^.

According to the Biopharmaceutics Drug Disposition Classification System, CBD is a class 2 drug, showing low water solubility and high permeability/metabolism depending on CYP3A4, CYP2C19, UGT1A9, and UGT2B7 enzymes^46^.

Clinical studies of CBD pharmacokinetics after oral administration were evaluated in different formulations such as capsules, solutions, and oromucosal preparations^47^.

Intravenous (IV) administration is the most usual administration route of antimicrobial therapy in hospitalized and critically ill patients, and CBD pharmacokinetics studies following IV administration have been studied in clinical trials^47^. CBD pharmacokinetics after a 20 mg IV dose was evaluated by a previous study, which showed clearance values of approximately 80 L/h and volume of distribution of 52 L in 70 kg individuals^48^. Thus, as an exercise of translational pharmacokinetics could be done using a classic equation and considering linear pharmacokinetics:

$${\text{Dose}}/\tau = {\text{CL}} \times {\text{Css}}$$

where τ is the dosing interval, CL is the total clearance, and Css is the steady-state mean plasma concentration. CBD is antibacterial against most Gram-positive cocci (GPC) (e.g., *Enterococcus* spp. and *Staphylococcus* spp.) and shows an in vitro MIC of 2 µg/mL or 4 µg/mL. However, CBD is not antibacterial against GNB. Nevertheless, the combination of CBD + polymyxin B (PB) is antibacterial against GNB, including MDR and XDR standard strains and clinical isolates. For most GNB, low concentrations of PB (MEAC, that are lower than PB MIC; and ≤ 2 µg/mL) allow CBD (≤ 4 µg/mL) to exert antibacterial activity against GNB (e.g., *Escherichia coli, Acinetobacter baumannii, Enterobacter cloacae*), highlighting PB-resistant GNB (e.g., *Klebsiella pneumoniae*). Considering data from the literature, to achieve these plasmatic concentrations, an IV administration of a CBD dose of approximately 2 g/12 h or 4 g/12 h would result in plasma exposure higher than MIC (Css) against GPC (alone) and GNB (in the combination CBD + PB).

Although these concentrations are target plasma concentrations and considering clearance values previously described, it could be possible to administer CBD doses to reach levels higher than the MIC, since single doses of approximately 6 g are described as well tolerated^46,49^.

To date, the pharmacokinetic data for CBD in the literature refer to another therapeutic context (pharmacoresistant epilepsy or refractory epilepsy treatment), which uses oral administration and different doses, determined by clinical studies specific for the therapeutic purpose described.

Our study shows the in vitro antibacterial activity of CBD, especially in combination with PB, suggesting potential repurposing of CBD as an antibacterial. To achieve this goal, research and development of new CBD formulations are needed to optimize the CBD pharmacokinetics to achieve higher serum concentrations from safe administration of the dosages required for antibacterial therapy. Our results, along with novel CBD formulations, present translational potential to be validated by future clinical studies for the purpose of treating bacterial infections.

In this context, optimization of CBD IV administration and pharmacokinetic parameters could be achieved using nanomaterial-based strategies, such as nanocarriers for increased solubility, stability, and efficacy for antibacterial therapy^50,51^. Thereby, future studies are needed regarding pharmacokinetics and pharmacodynamics, and safety and tolerability of CBD, alone or in the combination CBD + PB, in addition to MIC_50_ and MIC_90_ determination and probability of target attainment.

We highlight the promising translational potential of CBD repurposing as an antibacterial agent, mainly in the combination CBD + PB against GNB, for rescue treatment for life-threatening infections, highlighting against PB-resistant *K. pneumoniae*.

### Clinical perspectives of cannabidiol repurposing as antibacterial

Drug repurposing may represent a faster approach to identify new antimicrobials since preclinical and clinical parameters of these drugs are already established^52^. Thereby, CBD antibacterial activity might be considered for drug repurposing and should be evaluated in clinical studies (e.g., expanded access), initially against immediately life-threatening condition or serious infections^53^.

CBD could be evaluated in infections by:

i. XDR VISA (also optimizing pharmacokinetics/pharmacodynamics [PK/PD] parameters of clinical use of vancomycin);
ii. XDR *M. tuberculosis* (also using in combination with alternative tuberculostatic).

The combination CBD + PB might also be investigated in infection due to PB-resistant GNB, for which other antibiotics are ineffective:

i. Carbapenem-resistant *Enterobacteriaceae* (CRE) (e.g., *Klebsiella pneumoniae* carbapenemase [KPC] producers), ceftazidime-avibactam (CAZ-AVI)-resistant, and MDR/XDR;
ii. Carbapenem-resistant *A. baumannii* (CRAB) (for which CAZ-AVI is ineffective), and MDR/XDR;
iii. Pandrug-resistant (PDR) bacteria.

## Conclusion

CBD exhibited antibacterial activity against Gram-positive bacteria, *M. tuberculosis*, and LOS-expressing GND (*N. gonorrhoeae*, *N. meningitidis*, *M. catarrhalis*), but not against GNB. For most of the GNB studied, our results showed that the addition of low concentrations of PB (≤ 2 µg/mL) allow CBD (≤ 4 µg/mL) to exert antibacterial activity against GNB (e.g., *K. pneumoniae, E. coli, A. baumannii*), including PB-resistant GNB. CBD + PB also showed additive and/or synergistic effect against LOS-expressing GND. Our results show promising translational potential and suggest that CBD might be considered for drug repurposing, especially in the combination CBD + PB against GNB, highlighting PB-resistant *K. pneumoniae*.

## Methods

### Ethics declarations

This study was approved by the Ethics Committee of the School of Pharmaceutical Sciences of Ribeirao Preto (CAAE: 48834921.9.0000.5403-Protocol CEP/FCFRP nº580).

### Reagents

Cation-Adjusted Mueller Hinton II Broth (CAMHB) (BBL™, Becton Dickinson) and 96 wells microplates polystyrene, round bottom, non-treated, were used on all assays unless otherwise specified. For fastidious bacteria, such as *Streptococcus* spp*., Neisseria* spp*., Moraxella catarrhalis,* and *Haemophilus influenzae*, Cation-Adjusted Mueller Hinton II Broth (CAMHB) (BBL™, Becton Dickinson) supplemented with defibrinated horse blood and β-NAD, named MH-F, was used as recommended by EUCAST^31^.

Polymyxin B (PB) (United States Pharmacopeia) and ultrapure CBD (99.6%; BSPG-Pharm, Sandwich, UK) were used. As solvents, we used water for PB and methanol (Sigma-Aldrich) for CBD. Our previous standardization showed that methanol at concentrations ranging from 0.006 to 327.68 µL/mL on CAMHB is not antibacterial.

An aqueous solution of resazurin sodium salt (Sigma-Aldrich) was used to assess the metabolic activity and proliferation of bacterial cells, which were visually determined after bioreduction of the dye (blue) to resorufin (pink) by viable bacteria^25^.

### Investigation of CBD antibacterial activity against GP and GN bacteria

Antibacterial activity of CBD was investigated against a broad panel of different bacterial species, comprehending GP bacteria (13 different species; 21 strains), GN bacteria (30 different species; 73 strains), including type-strains, quality control strains, and clinical isolates (MDR and XDR strains, international high-risk clones, and also susceptible strains) (Supplementary Table 1)^27,29,30,54,55^.

Microdilution method was performed according to EUCAST recommendations for MIC determination, in agreement with the recommendations from the International Standards Organisation (ISO 20776-1 and ISO 20776-2)^26^. All MIC determination were performed in technical and experimental duplicates and, when the results were disparate, the MIC determination was repeated to confirm the results, considering the highest MIC value detected.

Two-fold serial dilution (256–0.5 µg/mL) of CBD were initially evaluated and MIC values were determined as the lowest concentrations of CBD that inhibit visible bacterial growth in broth culture medium. Polymyxin B and vancomycin were used as controls for GN and GP bacteria, respectively. In addition, ciprofloxacin was used as control for GND (*Neisseria* spp*., M. catarrhalis)*, and ampicillin for *S. pneumoniae* and *H. influenzae*^23^.

Beyond visual evaluation of growth inhibition, 30 µL of a 0.01–0.02% aqueous solution of resazurin sodium salt (Sigma-Aldrich) were added to each well of the microplate. Cell viability was assessed after 30–60 min for GNB and GPC and after 60–120 min for GND and *Enterococcus* species. This colorimetric step was additionally performed to allow better visualization of CBD antibacterial activity^25^.

CBD MBC was also evaluated for the GPC *Enterococcus faecalis* (ATCC 29212 and ATCC 51299), *E. faecium* (NCTC 7171 and ATCC 51559), and *S. aureus* (ATCC 29213 and ATCC 700699); as well as to the GND *N. meningitidis* (ATCC 13077), *N. gonorrhoeae* (ATCC 19424), and *M. catarrhalis* (ATCC 25238). The MBC method was performed after visual evaluation of growth inhibition by subculturing onto Mueller Hinton Agar or Mueller Hinton Agar with Blood (Difco™, Becton Dickinson) plates in the absence of CBD. MBC values were determined as the lowest concentration of CBD that prevents the growth of the bacterial colony-forming unit in solid culture medium.

### Investigation of CBD antibacterial activity against *M. tuberculosis*

We used the reference broth microdilution method to determine the CBD MIC against *M. tuberculosis* H37Rv (ATCC 27294) and also against rifampicin- and isoniazid-resistant *M. tuberculosis* CF86 (MDR clinical isolate), according to standard procedures^26^. Middlebrook 7H9 broth (Sigma-Aldrich) supplemented with 10% OADC (oleic acid, albumin, dextrose, catalase) and glycerol 0.4% was used and two-fold serial dilution (256–1 µg/mL) of CBD were evaluated.

Rifampicin and isoniazid were used as control (1–0.004 µg/mL). We used 96-well, polystyrene, flat-bottom microplates for the experiments. Each plate was incubated for seven days at 37 °C and 5% CO_2_. After incubation, 30 µL of 0.01% aqueous solution of resazurin sodium salt (Sigma-Aldrich) were added to each well of the microplate, and 24 h later the MIC was determined by fluorescence reading (excitation/emission 530/590 nm)^56^.

### Investigation of CBD antibacterial activity against GNB in the presence of efflux pump inhibitors

Efflux inhibition assays were performed to investigate CBD extrusion through efflux pumps from susceptible and resistant Gram-negative ESKAPE pathogens (*K. pneumoniae* ATCC 13883*, A. baumannii* ATCC 19606*, P. aeruginosa* ATCC 27853*, E. cloacae* ATCC 13047) as well as *E. coli* ATCC 25922 and 72H (MCR-1-producing clinical isolate) and *S. maltophilia* ATCC 13637 strains.

Broth microdilution method to determine CBD MIC was performed in the presence or absence of either PAβN (Sigma-Aldrich) (50 µg/mL), reserpine (Sigma-Aldrich) (50 µg/mL), or curcumin (256 µg/mL), in different assays^57,58^. We considered that a minimal threefold reduction in the MIC values in the presence of efflux pump inhibitors would be indicative of efflux-mediated resistance.

The colorimetric step using an aqueous solution of resazurin sodium salt (Sigma-Aldrich) was also performed to allow better visualization of CBD antibacterial activity.

### Investigation of the antibacterial activity of CBD in combination with PB against GN bacteria

#### Screening by broth microdilution method with a fixed concentration of CBD (256 µg/mL)

Screening of the antibacterial activity of the combination CBD + PB was evaluated against both PB-susceptible and PB-resistant GN bacteria, standard strains, and clinical isolates (13 species, 52 strains), including PB-susceptible and PB-resistant GNB (chromosomal-acquired and plasmid-mediated colistin-resistant [MCR-1] *E. coli*) and intrinsically-resistant GN bacteria (Tables 1 and 2).

To investigate the antibacterial activity of the combination CBD + PB, an initial screening was performed using the reference broth microdilution method with adaptations^26^: Two-fold serial dilutions (512–0.02 µg/mL) of PB were evaluated in the presence of 256 µg/mL of CBD (fixed concentration) in each well, including the bacterial growth control wells.

Furthermore, the antibacterial activity of the combination CBD (256 µg/mL, fixed concentration) + PB (two-fold dilution, 256–0.005 µg/mL) was also evaluated in the presence of PAβN (50 µg/mL), also including the bacterial growth control wells, for 7 species (13 strains). Cell viability assessment with resazurin was also performed as described above.

#### Confirmation by checkerboard assay

Checkerboard assays were performed to confirm the in vitro antibacterial activity and to assess the different proportions of each substance in the combination CBD + PB^59^. Final PB concentrations ranged from 0.01 to 512 µg/mL and CBD concentrations ranged from 2 to 256 µg/mL. For *P. aeruginosa* and plasmid-mediated colistin-resistant (MCR-1) *E. coli* strains, the assay was also performed in the presence of 50 µg/mL of PAβN. Confirmation of the antibacterial activity of the combination CBD + PB was performed against 22 selected strains (Tables 4 and 5).

The checkerboard assay usually aims to determine the fractional inhibitory concentration index (FICI) to categorize the combination (of two different substances, e.g., CBD and PB) as synergistic, additive, indifferent, or antagonist. FICI is calculated as: FICI = (CBD concentration in combination/MIC_CBD_) + (PB concentration in combination/MIC_PB_). FICI values are interpreted as: ≤ 0.5 (synergy), > 0.5–1 (additive), 1–4 (indifference) and > 4 (antagonism)^59^.

The antibacterial activity of the combination CBD + PB considered the best well(s) for which concentrations of PB (closest to 2 µg/mL or lower) combined to CBD (lowest concentrations) inhibited bacterial growth. Cell viability assessment with resazurin was also performed as described above.

#### Time-kill assay

Time-kill assays were used to evaluate the synergistic effect of the combination CBD + PB and were performed as previously described^60^ with slight modifications. The assays comprised 4 PB-resistant *K. pneumoniae* isolates, namely C9, L8, L28, and L29.

A bacterial suspension was prepared on McFarland's 0.5 scale (1.5 × 10^6^ colony forming units [CFU] per mL), added to MHB, and incubated at 37 °C under shaking until the logarithmic scale of bacterial growth (McFarland's 1.0 scale) is reached (around 4 h). This suspension was added to different MHB tubes containing the combination CBD (4 µg/mL) + PB MEAC; the combination CBD (2 µg/mL) + PB MEAC; PB MIC; PB MEAC; MHB without drugs or antibiotics as to bacterial growth control. After adding the suspension to each of the tubes (time zero), an aliquot was collected, diluted, and plated onto Mueller Hinton plates for subsequent CFU counting. Tubes were then incubated at 37 °C under shaking and the aliquot collection, dilution, and plating process was repeated after 1, 2, 4, and 6 h^60^. Three independent experiments were performed.

Results of the time-kill assays were analyzed by two different methods: (1) a statistical analysis comparing all treatments with a growth control; and (2) a more straightforward, traditional, and non-statistical method comparing CFU counts between the combination CBD + PB and PB alone^60^. For statistical testing of the former method, means were compared via ANOVA followed by Tukey’s test with statistical significance set to 0.05. All computations and graph plotting were performed with Prism 8 software (GraphPad Software, San Diego, CA, USA). For the latter method, combinations were considered synergistic when CBD + PB reduced CFU/mL by at least 2 log_10_ compared to PB alone^28^.

## Supplementary Information

Supplementary Information.
