# Supplementary material for: Potential cannabidiol (CBD) repurposing as antibacterial and promising therapy of CBD plus polymyxin B (PB) against PB-resistant gram-negative bacilli
Source: Sci Rep. 2022 Apr 19;12:6454. doi: 10.1038/s41598-022-10393-8 (PMC9018834; doi:10.1038/s41598-022-10393-8)
Supplement: Supplementary file 1 — Supplementary Information. [file 41598_2022_10393_MOESM1_ESM.docx]

**SUPPLEMENTARY MATERIAL:**

**Potential cannabidiol (CBD) repurposing as antibacterial and promising therapy of CBD plus polymyxin B (PB) against PB-resistant Gram-negative bacilli**

**Authors:** Nathália Abichabki^1^, Luísa V. Zacharias^1†^, Natália C. Moreira^1†^, Fernando Bellissimo-Rodrigues^2^, Fernanda L. Moreira^1^, Jhohann R. L. Benzi^1^, Tânia M. C. Ogasawara^1^, Joseane C. Ferreira^1^, Camila M. Ribeiro^3^, Fernando R. Pavan^3^, Leonardo R. L. Pereira^4^, Guilherme T. P. Brancini^1^, Gilberto Ú. L. Braga^1^, Antonio W. Zuardi^5,6^, Jaime E. C. Hallak^5,6^, José A. S. Crippa^5,6^, Vera L. Lanchote^1^, Rafael Cantón^7^, Ana Lúcia C. Darini^1^, Leonardo N. Andrade^1*^

^†^These authors contributed equally.

**Affiliations:**

^1^ School of Pharmaceutical Sciences of Ribeirão Preto (FCFRP) – University of São Paulo (USP), Department of Clinical Analyses, Toxicology and Food Science (DACTB) - Ribeirão Preto, SP, Brazil.

^2^ Ribeirão Preto Medical School (FMRP) – University of São Paulo (USP), Department of Social Medicine - Ribeirão Preto, SP, Brazil.

^3^School of Pharmaceutical Sciences (FCF) - São Paulo State University (UNESP), Department of Biological Sciences - Araraquara, SP, Brazil.

^4^ School of Pharmaceutical Sciences of Ribeirão Preto (FCFRP) - University of São Paulo (USP), Department of Pharmaceutical Sciences (DCF) - Ribeirão Preto, SP, Brazil.

^5^ Ribeirão Preto Medical School (FMRP) – University of São Paulo (USP), Department of Neurosciences and Behavioral Sciences - Ribeirão Preto, SP, Brazil.

^6^ National Institute of Science and Technology for Translational Medicine (INCT-TM), Conselho Nacional de Desenvolvimento Científico e Tecnológico (CNPq), Brasília, DF, Brazil.

^7^ Servicio de Microbiología. Hospital Universitario Ramón y Cajal and Instituto Ramón y Cajal de Investigación Sanitaria (IRYCIS), Madrid, Spain.

**Corresponding Author Information:**

Leonardo N. Andrade (leonardo@fcfrp.usp.br).

Universidade de São Paulo (USP, *University of Sao Paulo*)

Faculdade de Ciências Farmacêuticas de Ribeirão Preto (FCFRP, *School of Pharmaceutical Sciences of Ribeirao Preto*)

Departamento de Análises Clínicas, Toxicológicas e Bromatológicas (DACTB, *Department of Clinical Analyses, Toxicology and Food Science*)

Av. do Café, s/nº - Campus Universitário - CEP: 14040-903 - Ribeirão Preto - SP - Brasil
Tel.: +55 16 3315 4180

| **CBD MIC (µg/mL)** | **Strains** | **Characteristics; References** |
| --- | --- | --- |
|  | **GRAM-POSITIVE COCCI** |  |
| 2 | *Enterococcus faecium* NCTC 7171^T^ | Type strain |
|  | *Enterococcus faecium* ATCC 51559 | VRE, *vanA* |
| 4 | *Enterococcus faecalis* ATCC 29212 | Quality control strain |
|  | *Enterococcus faecalis* ATCC 51299 | VRE, *vanB* |
|  | *Enterococcus casseliflavus* ATCC 12361 | Susceptible strain |
|  | *Enterococcus gallinarum* ATCC 12359 | Susceptible strain |
|  | *Staphylococcus aureus* ATCC 29213 | Quality control strain |
|  | *Staphylococcus aureus subsp. aureus* ATCC 43300 | MRSA, *mecA* |
|  | *Staphylococcus aureus* N315 | MRSA/VSSA |
|  | *Staphylococcus aureus subsp. aureus* ATCC 700698 [Mu3] | MRSA, *mecA*; hVISA |
|  | *Staphylococcus aureus subsp. aureus* ATCC 700699 [Mu50] | MRSA, *mecA*; VISA |
|  | *Staphylococcus aureus subsp. aureus* ATCC BAA-976 | *msr(A)*-mediated macrolide-only resistance |
|  | *Staphylococcus aureus subsp. aureus* ATCC BAA-977 | inducible *erm(A)*-mediated resistance |
|  | *Staphylococcus lugdunensis* ATCC 43809 ^T^ | Type strain |
|  | *Staphylococcus saprophyticus subsp.saprophyticus* ATCC 53050^T^ | Type strain |
|  | *Staphylococcus epidermidis* ATCC 14990 ^T^ | Type strain |
|  | *Micrococcus luteus* CCT 2688 | Susceptible strain |
|  | *Rhodococcus equi* ATCC 6939 | Quality control strain |
| 32 | *Streptococcus pyogenes* ATCC 12344 ^T^ | Type strain |
|  | *Streptococcus pneumoniae* ATCC 49619 | Quality control strain |
| 64 | *Streptococcus agalactiae* ATCC 13813 ^T^ | Type strain |
|  | **MYCOBACTERIA** |  |
| 12.5 | *Mycobacterium tuberculosis* H37Rv ATCC 27294 ^T^ | Type strain |
| 25 | *Mycobacterium tuberculosis* CF86 | MDR Clinical isolate; Miyata *et al*, 2011^1^ |
|  | **GRAM-NEGATIVE DIPLOCOCCUS (GND)** |  |
| 64 | *Moraxella catarrhalis* ATCC 25238 ^T^ | Type strain |
| 128 | *Neisseria meningitidis* ATCC 13077 ^T^ | Type strain |
| 256 | *Neisseria gonorrhoeae* ATCC 19424 ^T^ | Type strain |
|  | **GRAM-NEGATIVE BACILLI (GNB)** |  |
| > 256 | *Haemophilus influenzae* ATCC 33391 ^T^ | Type strain |
|  | *Klebsiella pneumoniae subsp. pneumoniae* ATCC 13883 ^T^ | Type strain |
|  | *Klebsiella pneumoniae* ATCC BAA-1705 | ST 258; CRE/CPE, KPC-2, PB-susceptible |
|  | *Klebsiella pneumoniae* ATCC BAA-1706 | Negative control for KPC production |
|  | *Klebsiella pneumoniae* NCTC 13443 | CRE/CPE, NDM, PB-susceptible |
|  | *Klebsiella pneumoniae* C9 | ST 11; ESBL, CTX-M-2; PB-resistant [Δ *mgr*]; Palmeiro *et al*, 2019^2^ |
|  | *Klebsiella pneumoniae* D1 | ST 11; ESBL, CTX-M-2; Palmeiro *et al*, 2019^2^ |
|  | *Klebsiella pneumoniae* RP62 | ST 11; CRE/CPE, KPC-2; Andrade *et al*, 2014^3^ |
|  | *Klebsiella pneumoniae* L1 | Clinical isolate; PB-resistant |
|  | *Klebsiella pneumoniae* L2 | Clinical isolate; PB-resistant |
|  | *Klebsiella pneumoniae* L3 | Clinical isolate; PB-resistant |
|  | *Klebsiella pneumoniae* L5 | Clinical isolate; PB-susceptible |
|  | *Klebsiella pneumoniae* L8 | Clinical isolate; PB-resistant |
|  | *Klebsiella pneumoniae* L9 | Clinical isolate; PB-resistant |
|  | *Klebsiella pneumoniae* L12 | Clinical isolate; PB-susceptible |
|  | *Klebsiella pneumoniae* L13 | Clinical isolate; PB-resistant |
|  | *Klebsiella pneumoniae* L14 | Clinical isolate; PB-resistant |
|  | *Klebsiella pneumoniae* L15 | Clinical isolate; PB-resistant |
|  | *Klebsiella pneumoniae* L16 | Clinical isolate; PB-resistant |
|  | *Klebsiella pneumoniae* L17 | Clinical isolate; PB-resistant |
|  | *Klebsiella pneumoniae* L18 | Clinical isolate; PB-resistant |
|  | *Klebsiella pneumoniae* L19 | Clinical isolate; PB-resistant |
|  | *Klebsiella pneumoniae* L22 | Clinical isolate; PB-resistant |
|  | *Klebsiella pneumoniae* L26 | Clinical isolate; PB-resistant |
|  | *Klebsiella pneumoniae* L27 | Clinical isolate; PB-resistant |
|  | *Klebsiella pneumoniae* L28 | Clinical isolate; PB-resistant |
|  | *Klebsiella pneumoniae* L29 | Clinical isolate; PB-resistant |
|  | *Klebsiella pneumoniae* L30 | Clinical isolate; PB-susceptible |
|  | *Klebsiella pneumoniae* L31 | Clinical isolate; PB-resistant |
|  | *Klebsiella pneumoniae* L33 | Clinical isolate; PB-resistant |
|  | *Klebsiella pneumoniae* L34 | Clinical isolate; PB-resistant |
|  | *Klebsiella quasipneumoniae subsp. similipneumoniae*  ATCC 700603 | ESBL, SHV-18 |
|  | *Klebsiella oxytoca* CCT 0182 | Susceptible strain |
|  | *Klebsiella aerogenes* ATCC 13048 ^T^ | Type strain |
|  | *Enterobacter cloacae subsp. cloacae* ATCC 13047 ^T^ | Type strain |
|  | *Enterobacter cloacae subsp. cloacae* ATCC 23355 | Susceptible strain |
|  | *Escherichia coli* ATCC 25922 | Quality control strain, PB-susceptible |
|  | *Escherichia coli* ATCC 35218 | Quality control strain |
|  | *Escherichia coli* CTX-M-15 | ST 131; ESBL, CTX-M-15, PB-susceptible |
|  | *Escherichia coli* RP62T | Transconjugant azide-resistant producing KPC, PB-susceptible; Andrade *et al*, 2014^3^ |
|  | *Escherichia coli* 72H^#^ | Plasmid-mediated colistin-resistant (MCR-1); Fernandes *et al*, 2016^4^ |
|  | *Escherichia coli* NCTC 13846 | Plasmid-mediated colistin-resistant (MCR-1), Quality control strain |
|  | *Pantoea agglomerans* ATCC 33243 ^T^ | Type strain |
|  | *Cronobacter sakazakii* ATCC 29544 ^T^ | Type strain |
|  | *Citrobacter freundii* ATCC 8090 ^T^ | Type strain |
|  | *Serratia marcescens subsp. marcescens* ATCC 13880 ^T^ | Type strain, intrinsic PB-resistant |
|  | *Providencia rettgeri* ATCC 29944 ^T^ | Type strain, intrinsic PB-resistant |
|  | *Proteus mirabilis* ATCC 29906^T^ | Type strain, intrinsic PB-resistant |
|  | *Salmonella enterica subsp. enteric* (serovar Enteritidis)  ATCC 13076 | Quality control strain |
|  | *Shigella flexneri* ATCC 12022 ^T^ | Type strain |
|  | *Plesiomonas shigelloides* ATCC 14029 ^T^ | Type strain |
|  | *Hafnia alvei* ATCC 11604 | Susceptible strain |
|  | *Edwardsiella tarda* ATCC 15947 ^T^ | Type strain, intrinsic PB-resistant |
|  | *Morganella morganii* ATCC 8019 | Intrinsic PB-resistant |
|  | *Yersinia enterocolitica subsp. enterocolitica* ATCC 9610 ^T^ | Type strain |
|  | *Acinetobacter baumannii* ATCC 19606 ^T^ | Type strain, PB-susceptible |
|  | *Acinetobacter baumannii* 136 SP | ST 109; CRAB, OXA-23 and OXA-143; Clímaco *et al*, 2013^5^, PB-susceptible |
|  | *Acinetobacter baumannii* L7 | Clinical isolate; PB-susceptible |
|  | *Acinetobacter baumannii* L21 | Clinical isolate; PB-susceptible |
|  | *Acinetobacter baumannii* L25 | Clinical isolate; PB-susceptible |
|  | *Acinetobacter baumannii* L35 | Clinical isolate; PB-susceptible |
|  | *Pseudomonas aeruginosa* ATCC 27853 | Quality control strain, PB-susceptible |
|  | *Pseudomonas aeruginosa* HC103^ϕ^ | ST 277; SPM-1, PB-susceptible; Galetti *et al*, 2015^6^ |
|  | *Pseudomonas aeruginosa* L36 | Clinical isolate; PB-susceptible |
|  | *Pseudomonas aeruginosa* PAO1 | PB-susceptible |
|  | *Pseudomonas putida* ATCC 15175 | Susceptible strain |
|  | *Aeromonas hydrophila* ATCC 7966 ^T^ | Type strain |
|  | *Alcaligenes faecalis subsp. faecalis* ATCC 8750 ^T^ | Type strain |
|  | *Burkholderia cepacia* ATCC 25416 ^T^ | Type strain, intrinsic PB-resistant |
|  | *Stenotrophomonas maltophilia* ATCC 13637 ^T^ | Type strain, PB-susceptible |

**Supplementary Table 1.** CBD MIC results against bacterial strains studied. Antibacterial activity of CBD was investigated against a broad panel of different bacterial species, comprehending GP (13 different species; 21 strains) and GN (30 different species; 73 strains) bacteria and *M. tuberculosis* (2 strains), including type-strains, quality control strains, and clinical isolates (including MDR strains, international high-risk clones, and susceptible strains). *GP* Gram-positive, *GN* Gram-negative, *MDR* multidrug-resistant, *XDR* extensively drug-resistant, *CBD* cannabidiol, *MIC* minimal inhibitory concentration, *NCTC* National Collection of Type Cultures, *ATCC* American Type Culture Collection, ^T^ *type strain, VRE* vancomycin resistant *Enterococcus, MRSA* methicillin-resistant *Staphylococcus aureus, VISA* vancomycin intermediate resistant *Staphylococcus aureus, hVISA* heterogeneous vancomycin intermediate resistant *Staphylococcus aureus, CCT* Coleção de Culturas Tropical, Fundação Andre Tosello, Campinas – SP, Brazil, *ST* sequence type, *CRE* carbapenem-resistant *Enterobacteriaceae, CPE* carbapenemase-producing *Enterobacteriaceae,* *KPC* *Klebsiella pneumoniae* carbapenemase, *NDM* New Delhi metallo-beta-lactamase (carbapenemase), *ESBL* extended spectrum beta-lactamase, *CTX-M* Active on cefotaxime, first isolated at Munich, *SHV* Sulfhydryl reagent variable, *MCR* plasmid-mediated colistin resistant, *CRAB* carbapenem resistant *Acinetobacter baumannii, OXA* Oxacilinase (OXA-23 and OXA-143 are carbapenemases), *SPM* São Paulo Metallo-beta-lactamase (carbapenemase). PB-resistant Clinical isolates are not plasmid-mediated colistin-resistant (MCR-1). *Rhodococcus equi*, *Haemophilus influenzae,* and *Acinetobacter baumannii* are coccobacilli.


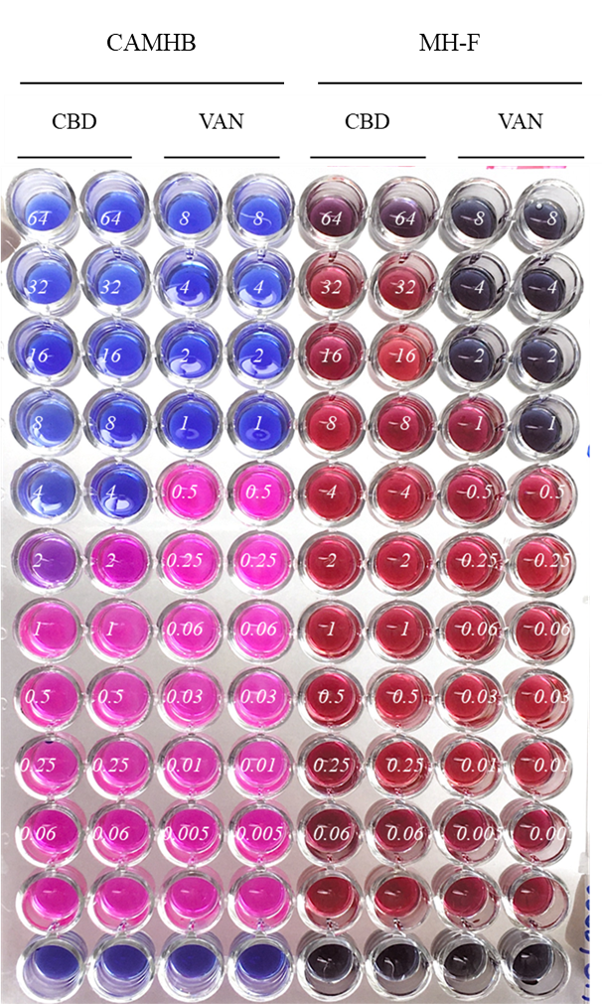


**Supplementary Figure 1.** CBD MIC results against *S. aureus* ATCC 29213 observed from broth microdilution assay using MH-F broth and using CAMHB. We observed higher CBD MIC (64 µg/mL) when the assay was performed using MH-F broth (5% lysed horse blood + 0.1% β-Nicotinamide adenine dinucleotide [β-NAD] 20 mg/mL), in comparison with standard protocol using CAMHB for *S. aureus* (CBD MIC = 4 µg/mL).

**
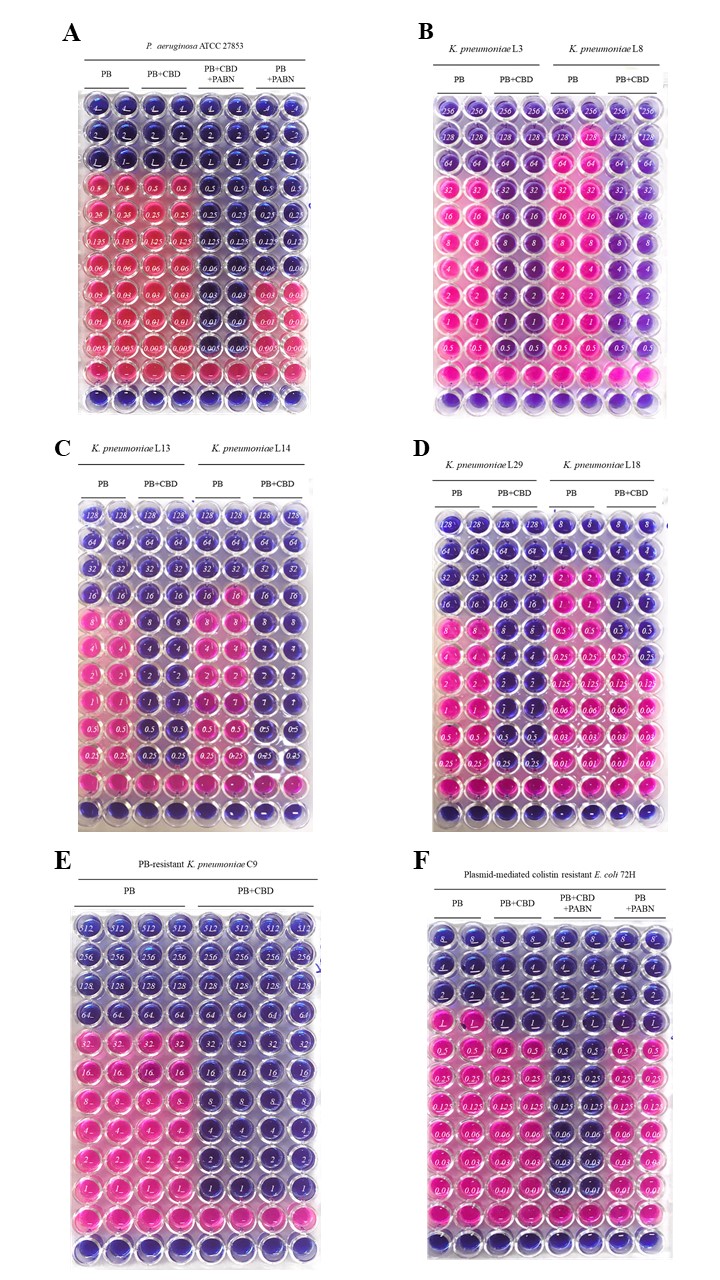

Supplementary Figure 2.** Representative screening assays results of antibacterial activity of the combination CBD + PB. The columns named “PB” are serial dilution of PB with no addition of CBD (PB MIC determination). The columns named “PB+CBD” are serial dilution of PB, plus a fixed concentration of CBD (256 µg/mL). The columns named “PB+CBD+PAβN” are serial dilution of PB, plus a fixed concentration of CBD (256 µg/mL) and a fixed concentration of PAβN (50 µg/mL). Finally, the columns named “PB+PAβN” are serial dilution of PB plus a fixed concentration of PAβN (50 µg/mL), with no addition of CBD. Blue wells show bacterial growth inhibition, while pink wells show bacterial growth. In each well, the numbers in italic refer to PB concentrations. Line “1 is MHB sterility control, while line “2” are bacterial growth control. Screening results of (**A**) PB-susceptible *P. aeruginosa* ATCC 27853, (**B**) PB-resistant *K. pneumoniae* L3 (left) and *K. pneumoniae* L8 (right), (**C**) *K. pneumoniae* L13 (left) and *K. pneumoniae* L14 (right), (**D**) *K. pneumoniae* L29 (left) and *K. pneumoniae* L18 (right), (**E**) PB-resistant *K. pneumoniae* C9, and (**F**) Plasmid-mediated colistin-resistant (MCR-1) *E. coli* 72H.


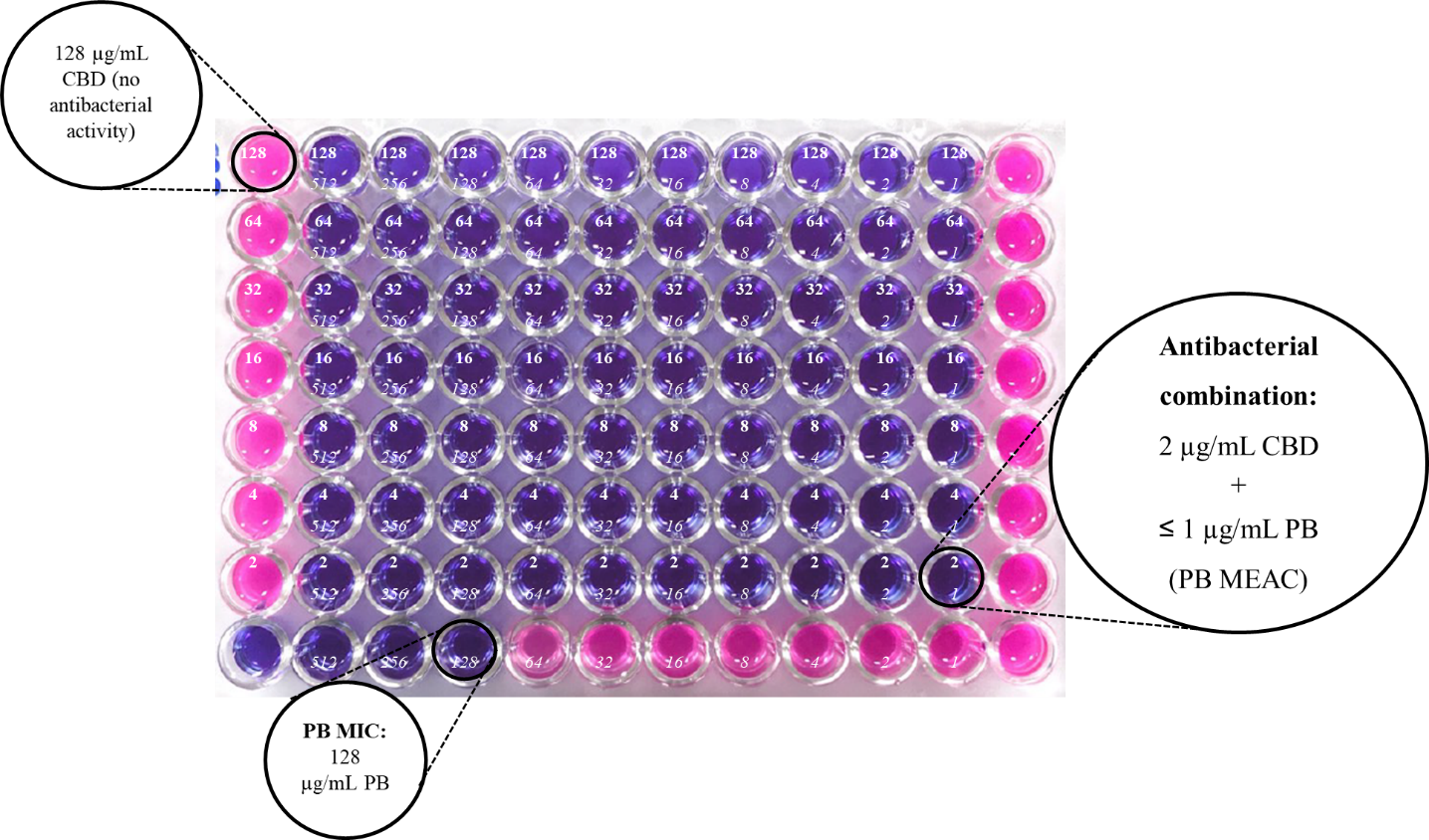


**Supplementary Figure 3.** Representative checkerboard assay results for PB-resistant *K. pneumoniae* L8, showing the concentrations of CBD and PB (PB minimal effective antibiotic concentration [MEAC]) required to the antibacterial activity of CBD in the combination CBD + PB. Blue wells are bacterial growth inhibition, while pink wells are bacterial growth. Serial dilution of CBD was performed horizontally (from the top to the bottom of the microplate), while serial dilution of PB was done vertically (from the left to the right of the microplate). In each well, the bold numbers of the top are referent to CBD concentrations, and the numbers in italic refer to PB concentration. Column “12” represents bacterial growth control, and the well “1H” is referent to the MHB sterility control.


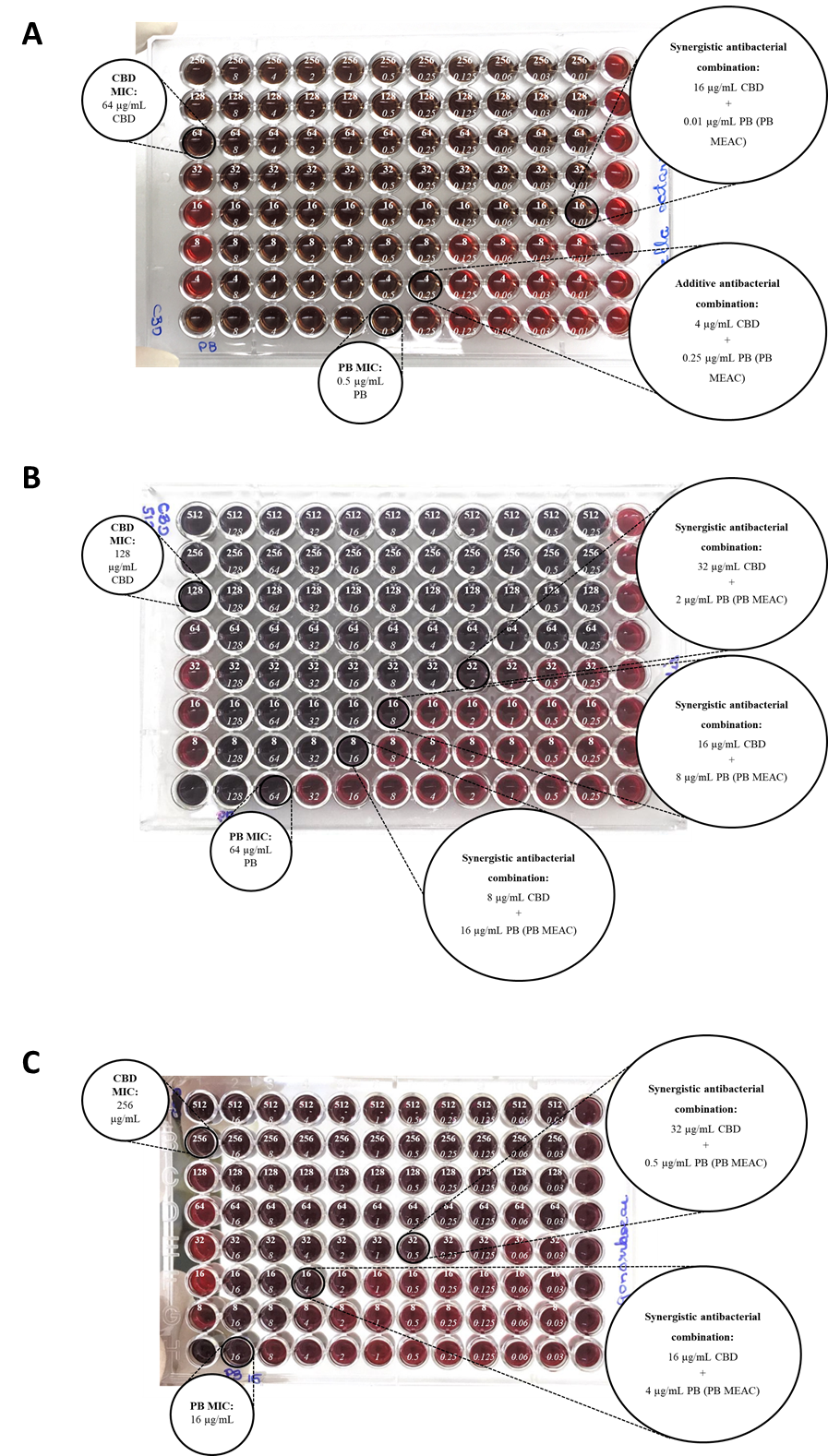


**Supplementary Figure 4.** Representative checkerboard assays results for GND showing the concentrations of CBD and PB (PB minimal effective antibiotic concentration [MEAC]) required to the antibacterial activity of CBD in the combination CBD + PB. Brown wells are bacterial growth inhibition, while red wells are bacterial growth. Serial dilution of CBD was performed horizontally (from the top to the bottom of the microplate), while serial dilution of PB was done vertically (from the left to the right of the microplate). In each well, the bold numbers of the top are referent to CBD concentrations, and the numbers in italic refer to PB concentration. Column “12” represents bacterial growth control, and the well “1H” is referent to the MHB sterility control. Checkerboard results for (**A**) *M. catarrhalis* ATCC 25238, (**B**) *N. meningitidis* ATCC 13077*,* and (**C**) *N. gonorrhoeae* ATCC 19424.

**References:**

1. Miyata, M. *et al.* Drug resistance in Mycobacterium tuberculosis clinical isolates from Brazil: Phenotypic and genotypic methods. *Biomed. Pharmacother.* **65**, 456–459 (2011).

2. Palmeiro, J. K. *et al.* Molecular Epidemiology of Multidrug-Resistant Klebsiella pneumoniae Isolates in a Brazilian Tertiary Hospital. *Front. Microbiol.* **10**, 1–11 (2019).

3. Andrade, L. N. *et al.* Expansion and evolution of a virulent, extensively drug-resistant (polymyxin B-resistant), QnrS1-, CTX-M-2-, and KPC-2-producing Klebsiella pneumoniae ST11 international high-risk clone. *J. Clin. Microbiol.* **52**, 2530–2535 (2014).

4. Fernandes, M. R. *et al.* Silent dissemination of colistin-resistant Escherichia coli in South America could contribute to the global spread of the mcr-1 gene. *Eurosurveillance* **21**, 1–6 (2016).

5. Clímaco, E. C. *et al.* Clonal complexes 104, 109 and 113 playing a major role in the dissemination of OXA-carbapenemase-producing Acinetobacter baumannii in Southeast Brazil. *Infect. Genet. Evol.* **19**, 127–133 (2013).

6. Galetti, R. *et al.* Genomic diversification and virulence features in SPM-1-producing Pseudomonas aeruginosa 13years later. *Diagn. Microbiol. Infect. Dis.* **82**, 179–180 (2015).
